# High-flow nasal cannula ventilation therapy for obstructive sleep apnea in ischemic stroke patients requiring nasogastric tube feeding: a preliminary study

**DOI:** 10.1038/s41598-020-65335-z

**Published:** 2020-05-22

**Authors:** Chien-Hui Ho, Chia-Ling Chen, Chung-Chieh Yu, Yao-Hung Yang, Chung-Yao Chen

**Affiliations:** 1 Department of Physical Medicine and Rehabilitation, Chang Gung Memorial Hospital, Keelung, Taiwan; 2 Department of Physical Medicine and Rehabilitation, Chang Gung Memorial Hospital, Linkou, Taiwan; 3 Graduate Institute of Early Intervention, College of Medicine, Chang Gung University, Taoyuan, Taiwan; 4 School of Medicine, College of Medicine, Chang Gung University, Taoyuan, Taiwan; 5 Division of Pulmonary, Critical Care and Sleep Medicine, Department of Internal Medicine, Chang Gung Memorial Hospital, Keelung, Taiwan

**Keywords:** Sleep disorders, Sleep disorders, Stroke, Stroke

## Abstract

Obstructive sleep apnea (OSA) is associated with increasing risk of recurrent stroke and mortality. Nasogastric tubes used by dysphagic stroke patients may interfere with nasal continuous positive airway pressure (CPAP) due to air leakage. This study was evaluated the effects and short-term tolerability of high-flow nasal cannula (HFNC) therapy for OSA in stroke patients with nasogastric intubation. The HFNC titration study was performed in post-acute ischemic stroke patients with nasogastric intubation and OSA. Then, participants were treated with HFNC therapy in the ward for one week. Eleven participants (eight males) who were all elderly with a median age of 72 (IQR 67–82) years and a body mass index of 23.5 (IQR 22.0–26.6) completed the titration study. The HFNC therapy at a flow rate up to 50~60 L/min significantly decreased the apnea-hypopnea index from 52.0 events/h (IQR 29.9–61.9) to 26.5 events/h (IQR 3.3–34.6) and the total arousal index from 34.6 (IQR 18.6–42.3) to 15.0 (IQR 10.3–25.4). The oxygen desaturation index was also significantly decreased from 53.0 events/h (IQR 37.0–72.8) to 16.2 events/h (IQR 0.8–20.1), accompanied by a significant improvement in the minimum SpO_2_ level. Finally, only three participants tolerated flow rates of 50~60 L/minute in one-week treatment period. Conclusively, HFNC therapy at therapeutic flow rate is effective at reducing the OSA severity in post-acute ischemic stroke patients with nasogastric intubation. Owing to the suboptimal acceptance, HFNC might be a temporary treatment option, and CPAP therapy is suggested after the nasogastric tube is removed.

## Introduction

Obstructive sleep apnea (OSA) is characterized by repetitive collapse of the pharyngeal airway during sleep, resulting in intermittent hypoxemia that is usually terminated by arousal from sleep, causing fragmentation of normal sleep^1^. Moderate-to-severe OSA was found to significantly increase cardiovascular risk, especially the risk of fatal or nonfatal stroke (relative risk, 2.02; 95% confidence interval 1.4–2.9) in a meta-analysis study^2^. Additionally, OSA has a negative effect on stroke outcome by worsening functional recovery and increasing the risk of recurrent stroke and mortality^3^ which is improved by proper treatment of OSA^4^. The most effective treatment for OSA is continuous positive airway pressure (CPAP). However, the compliance to CPAP in stroke patients was poor^5^, and the effects of CPAP on cerebral hemodynamics are uncertain^6,7^. CPAP has been reported to decrease cerebral blood flow velocity in acute stroke patients^8^. In addiction, the use of nasal CPAP has been reported to inhibit swallowing function^9^ and increase the risk of aspiration pneumonia^10^. Therefore, the benefits of CPAP in dysphagic stroke patients with OSA are questionable. Furthermore, nearly 30% of stroke patients were initially found to have dysphagia^11^, and most of them using nasogastric tube for nutrition support. The nasogastric intubation may cause air leakage during noninvasive ventilation therapy by nasal or oronasal masks^12^. Although CPAP by oral mask for OSA has been reported in some small-scale studies to be as effective as CPAP by nasal mask^13,14^, oral masks are not popular in clinical practice^15^ and have not been studied in stroke patients. Therefore, looking for alternative treatments for OSA in dysphagic stroke patients requiring nasogastric tube feeding is warranted.

High-flow nasal cannula (HFNC) oxygen therapy delivers adequately heated and humidified medical gas up to a flow rate of 60 L/minute and has the following physiological effects: anatomical dead space reduction, positive end-expiratory pressure (PEEP) effect, constant fraction of inspired oxygen, and adequate humidification^16^. By adequately conditioning the gas, HFNC ventilation therapy may enhance mucociliary function and accelerate the clearance of secretions^16^, which is an important concern for dysphagic stroke patients. The positive effect of HFNC therapy for OSA has been found in some small-scale studies^17–23^. McGinley *et al*. found that HFNC therapy decreased OSA severity in both adults^21^ and children^20^. In addition, the 2-week compliance of HFNC therapy for OSA was similar to that of CPAP therapy^23^. In a preliminary study, HFNC therapy was well tolerated and moderately decreased the apnea-hypopnea index (AHI) and the oxygen desaturation index (ODI) in 10 acute stroke patients^22^. The patients recruited in their study were all men with a relatively young age (56.8 ± 10.7 y/o) and mild stroke severity (HIHSS, 4 ± 4.3). In addition, central sleep apnea (CSA), which is usually noted in patients with acute stroke with unclear clinical significance and improve over time^3^, was not excluded in their study. Therefore, their results cannot be generalized to more severe stroke patients with OSA. Furthermore, a low flow rate of 18 L/min was selected to guarantee comfort, and a higher flow rate was suggested to yield a better reduction in sleep apnea severity^22^.

The main objective of this study was to evaluate the effects and short-term tolerability of HFNC therapy at the therapeutic flow rate for OSA in dysphagic stroke participants with nasogastric intubation.

## Materials and Methods

### Participants

This study recruited post-acute (one week after the stroke onset) dysphagic ischemic stroke patients, with nasogastric intubation admitted consecutively to the Rehabilitation ward of a teaching hospital. The age of enrolled patient was above 20 years old. After a full clinical examination and neuroimaging studies, they were diagnosed with ischemic stroke and the diagnosis of dysphagia was made by a speech pathologist. Participants with congestive cardiac failure, severely compromised consciousness, chronic obstructive pulmonary disease, history of intracranial hemorrhage or malignance, and unstable medical and neurological conditions such as severe infection or uncontrolled diabetes mellitus were excluded from this study. Participants with CSA or other sleep disordered breathing were also excluded.

**Figure 1:**
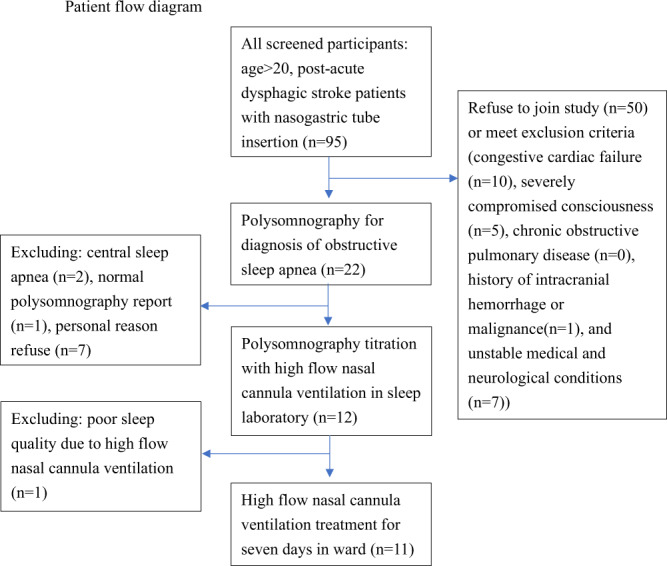
Patient flow diagram.

The study protocol (Fig. 1) was approved by the Institutional Review Board of Chang Gung Medical Foundation. The committee that approved the research confirmed that all methods were performed in accordance with relevant guidelines and regulations, and that informed consent was obtained from all participants and/or their legal guardians. This study was registered in the WHO International Clinical Trial Registry Platforms with ID number of NCT04173767 on 22/11/2019.

### Clinical evaluation

Upon admission, a comprehensive history of stroke risk factors and demographic data was taken. Body mass index and the Barthel Index (BI)^24^ were measured on the same day as polysomnography (PSG) examination. The BI was used to evaluate the functional outcomes in stroke patients and might represent stroke severity.

### Polysomnography

The PSG was tested with a Compumedics Profusion system (Australia). It was conducted at the sleep center from 10:00 pm to 7:00 am. The American Academy of Sleep Medicine (AASM) scoring manual version 2.0.3 was used to diagnose sleep apnea^25^. The PSG comprised six electroencephalography channels, thoracic and abdominal movement sensors (inductance plethysmography), an oxyhemoglobin saturation detector (finger pulse oximetry), a chin and bilateral anterior tibial surface electromyogram, an electro-oculogram, an electrocardiogram and nasal and oral airflow sensors (nasal pressure cannula and oronasal thermistor). Because the nasal and oral airflow sensors are unreliable while on HFNC, the respiratory inductance plethysmography sum (RIPsum) without calibration was used for scoring apnea and hypopnea events in accordance with AASM’s alternative criteria. Apnea was defined as a peak signal excursion drop ≥ 90% for at least 10 seconds; hypopnea was defined as a reduction in the peak signal excursion ≥ 30% for ≥ 10 seconds with either an arousal or oxygen desaturation ≥ 3%; and the number of times per hour of sleep that the blood’s oxygen saturation level drops by ≥ 3% from baseline was defined as the oxyhemoglobin desaturation index (ODI). As the RIPsum method tend to underestimate apnea events, especially the obstructive apnea events^26^, the AASM mentioned that previous studies^27,28^ using this method usually analyzed the combination of hypopneas and apneas^29^. Therefore, this study did not analyze apneas and hypopneas separately. The diagnosis of OSA was made when >50% of respiratory events were obstructive or mixed type in PSG. The diagnosis of CSA was made when ≥50% of respiratory events were the central type. One author, a board-certified somnologist, visually scored the PSGs.

### High-flow nasal cannula ventilation titration

**Figure 2:**
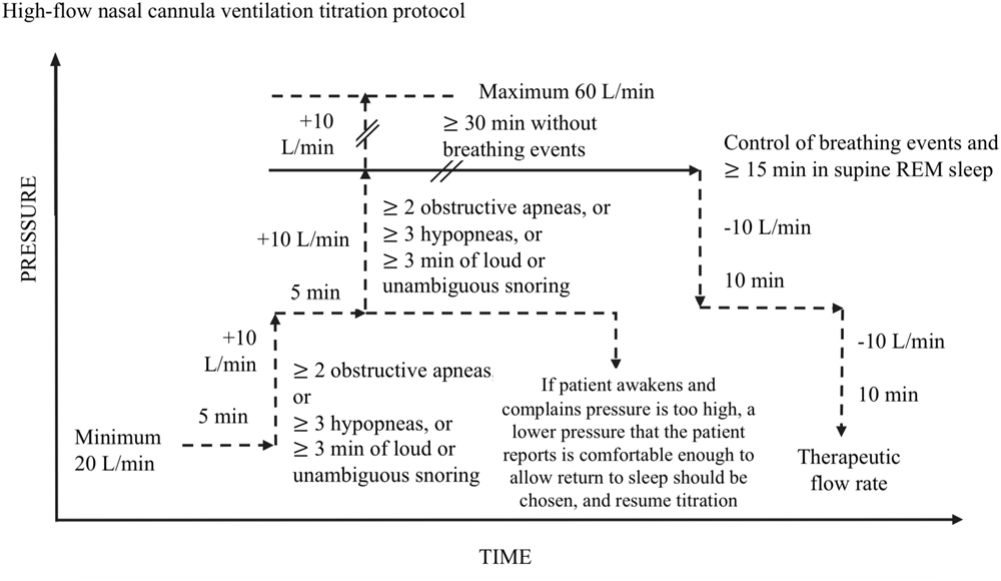
High-flow nasal cannula ventilation titration protocol.

Once OSA was diagnosed, a HFNC ventilation titration study was performed within two weeks in the sleep center with a simultaneously PSG study. An air compressor (AIRVO^TM^ 2, Fisher & Paykel Healthcare, Auckland, New Zealand) delivered room air at a high flow rate through the nasal cannula (Optiflow^TM^ system). A delivery heating tube (900PT500) and humidifier included in the AIRVO^TM^2 device maintained a temperature of 37 °C and absolute humidity of 44 mg/L. The rule of flow rate adjustment is similar to CPAP titration guideline^30^ (Fig. 2). The initial flow rate was 20 L/minute and FiO2 was maintained at room air level of 21% and was then titrated gradually in 10 L/min increments when more than two apnea, three hypopnea, or three minutes of loud or unambiguous snoring were found. The time interval between each increase in the flow rate was at least five min. AIRVO^TM^ 2 has a maximum flow rate of 60 L/minute. The titration continued until reach therapeutic flow rate, when the maximum flow was reached or the participant could not tolerate it or no respiratory events (≥2 obstructive apneas, or ≥ 3 hypopneas, or ≥ 3 min of loud or unambiguous snoring) were noted. After the therapeutic flow rate was reached, OSA severity and sleep parameters were measured.

### High-flow nasal cannula ventilation therapy

The next day after the titration study, participants began the HFNC treatment for one week in the ward. The HFNC flow rate was kept constant without ramping every night and the beginning flow rate on the first night was set at 20 L/minute. On the following nights, the flow rate was increased in 10 L/minute increments per night if participants could sleep with HFNC more than four hours until the therapeutic flow rate determined by the titration study was reached. The reason why participants could not tolerate HFNC for more than four hours would be recorded and corrected if possible and the flow rate will be kept in the last tolerate flow rate.

### Statistical analysis

Data are shown as the median and interquartile range (IQR), if not otherwise specified. The Wilcoxon Signed Ranks test was performed to compare differences in polysomnographic indices between the diagnostic and HFNC titration studies. Statistical power analyses with an effect size of 1, 2-tailed α of 0.05, and power of 0.8 were conducted, using G-Power version 3.1.9.5 software (Heinrich-Heine-University, Düsseldorf, Germany), which resulted in 11 subjects. As participants need nasogastric tube feeding, the expected flow rate of HFNC therapy to achieve therapeutic effect would be higher than that in subjects without nasogastric intubation. Considering a possible 33% drop-out rate because of intolerance to HFNC therapy^22^, we decided to recruit at least 17 patients. Good response to HFNC therapy was defined as an AHI decrease of more than 50% of the baseline AHI. A p value < 0.05 was considered statistically significant. Statistical analyses were performed using SPSS software (IBM SPSS Statistics 25, SPSS Inc., Chicago, IL).

## Results

There were 22 participants enrolled in this study. However, seven of them refused the HFNC titration study due to discomfort during the initial PSG study and two of them were excluded due to CSA or sleep-related hypoventilation due to a medical disorder diagnosed by the initial PSG study. One participant was excluded because of the normal PSG report. Twelve participants received a HFNC titration study, but one of them showed very poor sleep quality in the titration test, which was terminated prematurely without useful results.

The remaining 11 participants (8 males and 3 females) who completed the titration study were all elderly with a median age of 72 (IQR: 67 to 82) years and a BMI of 23.5 (IQR: 22.0 to 26.6). They were severely disabled^31^ with a Barthel index of 20 (IQR: 10 to 35) and were in the post-acute stroke phase at an average of 1.3 months (IQR: 1.1 to 2.8) after stroke onset. Brainstem stroke was found in only one of them. Severe OSA with an AHI of 52.0 events/h (IQR 29.9 to 61.9)) and sleep efficiency of 68.6% (IQR 39.7 to 76.2) was found in the initial PSG study. The final flow rate of the titration study was 60 L/min in eight participants and 50 L/min in three participants.

The results of initial PSG and HFNC therapy at the therapeutic flow rate are shown in Table 1. After using HFNC therapy at the therapeutic flow rate, the AHI significant decreased to 26.5 events/h (IQR 3.3 to 34.6), with a reduction rate of 49%. The ODI significantly decreased from 53.0 events/h (IQR 37.0 to 72.8) to 16.2 events/h (IQR 0.8 to 20.1), with a reduction rate of 69.4%, which was accompanied by a significant improvement in the minimum SpO_2_ level. Despite the significant reduction in the respiratory and total arousal index, the sleep architecture parameters including sleep efficiency did not change significantly after HFNC therapy.

**Table 1:** The result of polysomnography in baseline and HFNC therapy at the therapeutic flow rate.

	Baseline	HFNC	*p value*
AHI	52.0 (29.9–61.9)	26.5 (3.3–34.6)	0.026*
Minimum SpO_2_	78.0 (74.0–80.0)	88.0 (82.0–92.0)	0.009*
Mean SpO_2_	94.0 (92.8–94.3)	95.0 (93.0–96.0)	0.106
ODI	53.0 (37.0–72.8)	16.2 (0.8–20.1)	0.007*
Sleep efficiency %	68.6 (39.7–76.2)	70.7 (43.1–82.2)	1
REM %	8.7 (3.5–9.0)	8.3 (0–35.5)	0.374
Deep sleep %	23.4 (14.4–31.1)	24.0 (10.1–35.7)	0.638
Spontaneous arousal index	6.3 (2.1–11.8)	8.7 (5.8–14.4)	0.386
Respiratory arousals index	28.1 (13.5–30.5)	4.1 (2.5–12.3)	0.005*
Total arousal index	34.6 (18.6–42.3)	15.0 (10.3–25.4)	0.022*

AHI: apnea-hypopnea index, ODI: oxyhemoglobin desaturation index, SpO2: oxyhemoglobin saturation by pulse oximetry, REM: rapid eye movement, HFNC: high-flow nasal canula.

Data are median (IQR).

*p < 0.05.

**Figure 3:**
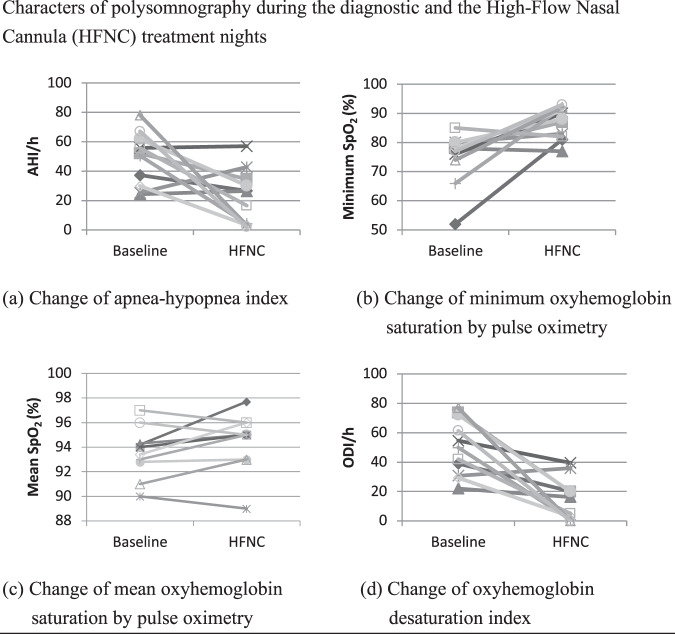
Characters of polysomnography during the diagnostic and the High-Flow Nasal Cannula (HFNC) treatment nights.

However, the effect of HFNC therapy on OSA severity was heterogeneous among participants, as shown in Fig. 3. Six (55%) out of 11 participants were good responders with more than a 50% reduction in the AHI after HFNC therapy. The polysomnographic, demographic, and anthropometric differences between good and poor responders are shown in Table 2. The good responders tended to have a higher AHI (58.8 events/h (IQR 45.4 to 69.9) vs 37.3 events/h (IQR 24.8 to 53.9)) compared to the poor responders. Otherwise, they were similar with respect to the Barthel index, BMI, SpO_2_ level, and sleep efficiency.

**Table 2:** Comparison of anthropometric and clinical data between good and poor responders.

	Total	Good responders	Poor responders	*P value*
Case number	11	6	5	
Male	8	4	4	0.576
Smoking	4	2	2	0.652
Hypertension	10	5	5	0.545
Diabetes Mellitus	2	1	1	0.727
Dyslipidemia	6	4	2	0.392
Recurrent stroke	4	2	2	0.652
Dysphagia	10	5	5	0.545
Atrial fibrillation	5	2	3	0.392
Age	72 (67–82)	69.7 (63.3–76.9)	72.6 (70.8–82.3)	0.429
Barthel index	20.0 (10.0–35.0)	17.5 (13.8–33.8)	30.0 (5.0–37.5)	0.931
BMI	23.5 (22.0–26.6)	22.8 (15.7–26.7)	23.7 (23.0–26.7)	0.429
AHI	52.0 (29.9–61.9)	58.8 (45.4–69.9)	37.3 (24.8–53.9)	0.082
ODI	53.0 (37.0–72.8)	55.8 (40.7–73.5)	47.6 (32.5–67.0)	0.429
Mean SpO_2_	94.0 (92.8–94.3)	93.2 (92.4–96.3)	94.0 (92.0–94.3)	0.931
Minimum SpO_2_	78.0 (74.0–80.0)	77.5 (72.0–81.3)	78.0 (64.0–79.0)	0.792
Sleep efficiency %	68.6 (39.7–76.2)	68.9 (37.5–76.6)	68.6 (40.5–79.0)	0.662
REM %	8.7 (3.5–9.0)	8.9 (4.9–11.1)	5.7 (1.6–11.2)	0.329
Deep sleep %	23.4 (14.4–31.1)	19.6 (14.0–31.9)	23.4 (11.2–42.9)	0.792

BMI: body mass index, AHI: apnea-hypopnea index, SpO2: oxyhemoglobin saturation by pulse oximetry, REM: rapid eye movement, ODI: oxyhemoglobin desaturation index.

Data are median (IQR). *P* value: comparison between good and poor responders.

Three of the 11 participants did not complete the 7-day therapy course due to intolerance. The final flow rate at the 7^th^ therapy night was 30 L/min (3 participants), 40 L/min (2 participants), 50 L/min (2 participants) and 60 L/min (1 participant).

## Discussion

To the best of our knowledge, this is the first study to evaluate the effect of HFNC therapy for OSA on post-acute ischemic stroke patients with nasogastric intubation and HFNC therapy at the therapeutic flow rate significantly improved OSA severity. One major strength of this study is that it tested the acceptance of HFNC therapy in the ward setting for one week. Although HFNC therapy by ramping the flow rate gradually up to 50~60 L/minute decreases arousals and does not deteriorate sleep efficiency in a well-controlled sleep center, its tolerability in the ward setting is suboptimal, and further study to improve patient acceptance is indicated.

HFNC therapy at the therapeutic flow rate effectively reduces the OSA severity. Previous studies suggested that the possible mechanism of HFNC therapy for OSA is the alleviation of upper airway obstruction by increasing nasopharyngeal pressure^16,21^. There is no absolute contraindication of HFNC therapy^16^. However, the influence of nasogastric intubation on the application of HFNC therapy has not yet been reported. The nasal cavity was found to account for two-thirds of the total airway resistance^32^. Nasogastric intubation was found to significantly increase nasal resistance and total airway resistance in infants^33^. The relatively large volume of the nasogastric tube to the volume of the nasal cavity might be the main reason that a higher flow rate up to 50~60 L/min is required to significantly improve OSA severity. The 49% reduction in the overall AHI at an therapeutic flow rate is nearly threefold the night-to-night variability in OSA severity of 13%^34^ and is clearly better than previous studies on normal subjects^18^ at 20 L/min flow rate (23.9% reduction, from 22.6 ± 15.6 to 17.2 ± 13.2 events/h) and male acute stroke patients without nasogastric intubation^22^ at 18 L/min flow rate (23.8% reduction, from 40.4 ± 25.7 to 30.8 ± 25.7 events/h). Despite the general therapeutic effect of HFNC therapy observed in this study, the individual response is heterogeneous, and stroke patients with more severe OSA tend to respond better to HFNC therapy. Previous studies in children^19^ and middle-aged adults^18^ suggested that hypopnea-predominant OSA responded better to HFNC therapy. Nilius *et al*. recruited 54 middle-aged, male-predominant adults with a mean BMI of 28 and moderate hypopnea-predominant OSA and found that the response rate was associated with a greater hypopnea rate in a dose-dependent fashion^18^. Given the employment of the RIPsum method, results of this study did not include separate analysis of hypopnea and apnea events and cannot be compared with their findings^18^.

The degree of intermittent hypoxia due to OSA is significantly improved after HFNC therapy. In our study, the improvement in the ODI was more obvious than the improvement in the AHI (the reduction rates of the ODI and AHI were 69.4% and 49%, respectively). Our results are in agreement with previous studies in acute stroke patients, which found that HFNC therapy at an 18 L/minute flow rate decreased the ODI moderately from 40.7 ± 28.4 to 31 ± 22.5 events/h, thus a 23.8% reduction in the ODI, while the mean SpO_2_ level remained unchanged^22^. In addition, the minimum SpO_2_ level was significantly improved from 78.0% (IQR 74.0 to 80.0) to 88.0% (IQR 82.0 to 92.0) after HFNC therapy. Therefore, HFNC therapy at a high flow rate above 50 L/min not only improved the frequency of oxygen desaturation but also the severity of oxygen desaturation. The minimum SpO_2_ level was found to be independently associated with interleukin-6 and C-reactive protein levels in OSA patients^35^. Nocturnal hypoxia is associated with atrial fibrillation (Af)^36^, neurological deterioration^37^ and cerebral white matter hyperdensities^38^ in stroke patients. Reducing the intermittent hypoxia in dysphagic stroke patients with OSA using HFNC therapy may improve comprehensive stroke treatment.

HFNC therapy with a gradually increasing flow rate up to 50~60 L/min performed at the sleep center decreases arousal events and does not deteriorate sleep architecture and efficiency in post-stroke dysphagic patients with OSA. Previous studies of middle-aged adults^23^ with flow rates up to 35 L/min, acute stroke patients^22^ with flow rates of 18 L/min and children^19^ with flow rates up to 50 L/min also found that HFNC therapy did not deteriorate sleep efficiency. The effect of HFNC on arousal is inconclusive as two studies using a relatively low flow rate (18^22^ and 20^21^ L/min) reported a decrease in the arousal index and one study using a relatively high flow rate (35^23^ L/min) reported the same arousal index. Although the discomfort due to the high airflow rate slightly increased the spontaneous arousal index in this study, the respiratory and total arousal index was significantly decreased by HFNC therapy which is in agreement with studies recruiting acute stroke patients^22^.

Although HFNC therapy by ramping the flow rate gradually up to 50~60 L/minute does not deteriorate sleep efficiency in a well-controlled sleep center, its acceptance in the ward setting is suboptimal. Therefore, the significant therapeutic effect of HFNC in the sleep center cannot be generalized to the ward setting. Most studies focusing on HFNC therapy for OSA evaluated only one-night therapeutic effects and tolerability^18,19,21,22^ and very few studies reported short-term adherence. Sowho *et al*. compared the 2-wk adherence of home-based CPAP and HFNC therapy at flow rates ranging from 10–35 L/min for mild OSA adults and found that the average HFNC therapy usage time was 3.6 ± 1.6 h/night and the percentage of nights with HFNC usage more than 4 h was approximately 50%^23^. Amaddeo *et al*. studies 8 children with OSA and found poor one-month compliance in three (60%) out of five children who received HFNC at 20 L/min^17^. In this study, three (27.2%) out of 11 participants could not complete a 7-day treatment course because of nasal congestion, noise and difficulty initiating sleep. The nasal congestion might be related to the humidity and temperature setting of the HFNC therapy. Only two participants were treated at 50 L/min and one participant was treated at 60 L/min on the 7^th^ night of HFNC therapy. Difficulty in initiating sleep was noted by others because the flow rate at the beginning of therapy was too high to tolerate. However, it requires many resources and is impractical to manually ramp up the flow rate of HFNC gradually and adjust the corresponding temperature and humidity of the gas in the ward according to participants’ response every night. The development of an overnight HFNC ramping protocol that automatically controls the flow rate, humidity and temperature is suggested to improve the patient’s tolerance to a high flow rate in the ward setting or even in the home setting.

There are several limitations of this study. First, the small sample size in this preliminary study makes further analysis of predictors for treatment response such as stroke topography or etiology difficult. Most studies regarding HFNC therapy for OSA were small-scale. Further studies with larger sample sizes are suggested to elucidate this issue. Second, the apnea and hypopnea events were not analyzed separately because of the employment of RIPsum method in this study. Caution should be taken in direct comparison between studies using different sensors for airflow detection. Third, participants in this study were highly selected, which made its generalizability to ischemic stroke patients with different stroke topography, etiology and severity limited. Lastly, the potential bias for underscoring PSG in HFNC titration study might happened since titration was performed and scored by one of our authors.

## Conclusion

HFNC therapy at therapeutic flow rate is effective at reducing OSA severity in post-acute ischemic stroke patients fed with a nasogastric tube, especially when the severity of OSA is high. Owing to the suboptimal acceptance, HFNC therapy might be a temporary treatment option for OSA, and nasal CPAP therapy is suggested after dysphagia improves and nasogastric intubation is removed. In addition, the development of an automatic ramping protocol for HFNC therapy is recommended to improve therapeutic effect.
